# A pragmatist approach to clinical ethics support: overcoming the perils of ethical pluralism

**DOI:** 10.1007/s11019-018-09882-3

**Published:** 2019-01-25

**Authors:** Giulia Inguaggiato, Suzanne Metselaar, Rouven Porz, Guy Widdershoven

**Affiliations:** 1 Department of Medical Humanities, EMGO+ Institute for Health and Care Research, VU University Medical Centre (VUmc), Amsterdam, The Netherlands; 2 Clinical Ethics Unit, Bern University Hospital ‘Inselspital’, Inselgruppe AG, Bern, Switzerland

**Keywords:** Pragmatism, Clinical ethics support, Ethical pluralism, Moral case deliberation, Dilemma method

## Abstract

In today’s pluralistic society, clinical ethics consultation cannot count on a pre-given set of rules and principles to be applied to a specific situation, because such an approach would deny the existence of different and divergent backgrounds by imposing a dogmatic and transcultural morality. Clinical ethics support (CES) needs to overcome this lack of foundations and conjugate the respect for the difference at stake with the necessity to find shared and workable solutions for ethical issues encountered in clinical practice. We argue that a pragmatist approach to CES, based on the philosophical theories of William James, John Dewey, and Charles Sanders Peirce, can help to achieve the goal of reaching practical solutions for moral problems in the context of today’s clinical environment, characterized by ethical pluralism. In this article, we outline a pragmatist theoretical framework for CES. Furthermore, we will show that moral case deliberation, making use of the dilemma method, can be regarded an example of a pragmatist approach to CES.

The most popular approaches to bioethics, namely principlism and casuistry, generally regard moral deliberation as a “justificatory” process (Pamental 2013, p. 727) meant to ethically validate health care decisions on the of basis of “common paradigms” and “uncontroversial moral touchstones” (Turner 2003, p. 113). Hence, in practice, moral reasoning in health care is mostly guided by a-priori principles, such as ‘respect for autonomy’, ‘beneficence’ and ‘justice’. However, by presupposing the existence of undisputed, i.e. stable and consistent, “mid-level prima facie” principles (Ainslie 2002, p. 3), these approaches fail to sufficiently acknowledge the existence of fundamental ethno-cultural and life style differences among stakeholders (Turner 2003, p. 109). In a pluralistic setting such as our contemporary society, different and sometimes conflicting moral values and principles influence views on ‘good care’.

In this context, moral judgments and moral reasoning cannot sufficiently be justified on the basis of a given set of shared fundamental principles, values or rules that serve as a more or less uncontroversial basis of ethics (Engelhardt 2011; Hester 2003, p. 549). Each and every situation might entail a fundamental diversity that should not be overlooked (Chattopadhyay and De Vries 2012, p. 640). Yet, clashes between different moral backgrounds need to be solved in order to reach shared decisions on how to act.

If we let go of the existence of a broad universal consensus on basic moral notions, we might slip into moral scepticism or radical relativism, precluding moral judgments on what is right or wrong, and denying the existence of moral expertise (McClimans and Slowther 2016). This raises the question whether clinical ethics support (CES), in its present state, can deal with the challenges of ethical pluralism, without falling into moral scepticism. The question is how to provide CES in a pluralistic context, and support health care givers in reconciling the clashes between different moral backgrounds, given the absence of shared foundational moral principles.

A similar question has been addressed by American pragmatists in another context and era. At the end of the nineteenth century, the pioneers of classical American pragmatism dedicated themselves to exploring how to defeat scepticism while embracing a fallibilist approach to our principles and beliefs (Putnam 1995). They turned their back to absoluteness and metaphysical truths, and instead of looking for general, fundamental principles, they argued that our ideas have to be regarded as “tools” arising from a singular situation, and responding to a specific problem (Dewey 1957, p. 169). According to pragmatism, morality is a dynamic process aiming to creatively respond to a continuously changing reality (Dewey 1957, pp. 95–97, 144–145); in their view, morality cannot be seen as a process in which we apply fixed and presupposed moral notions to a specific situation, since all situations need to be treated in response to their particularities (Dewey 1957, pp. 169–70).

Various authors have proposed a pragmatist approach to bioethics (Arras 2002; Cooke 2003; Fins et al. 1997, 1998; Hester 2003; Keulartz et al. 2002; McGee 2003; Tollefsen 2000; Mallia and ten Have 2005). As Wolf (1994) argues, pragmatism corresponds with the agenda of a (at that time) new approach to bioethics arising at the end of the twentieth century, abandoning the traditional “principle or rule-driven” (ivi, p. 399) approach in favour of a more empirical, bottom-up approach, focusing on the actual needs and characteristics of patients and stakeholders, also paying more attention to gender-, race-, ethnicity-, and religion-related issues. “John Dewey, William James, and Charles Sanders Peirce have come to visit the clinic and find much to criticize” (Wolf 1994, p. 398).

Around the turn of the century, two relevant contributions to bioethics from a pragmatist perspective were published. The first is presented in the anthology edited by Glenn McGee in 1999, and addresses theoretical issues, focusing on topics such as end of life and genetics from a pragmatists perspective. The second, articulated by Miller, Fins and Bacchetta, presents a “clinical pragmatist” approach based on Dewey’s work, providing a “process model” to guide practitioners in solving specific moral problems they encounter in everyday practice (Miller et al. 1996, p. 47). The aim of this article is to explore how a pragmatist approach can provide tools for the practice of CES, and to consider how this approach may translate into an actual method to be used in practice. We will expand upon the initiatives just mentioned, both theoretically and practically. Theoretically, we will refer not only to the work of Dewey, but also to that of Charles Sanders Peirce and William James. Practically, we will present an example of an approach to CES that is in line with pragmatism, and can be viable in a pluralistic setting.

We will argue that pragmatism is valuable for present-day CES as it contains four core elements which enable us to (1) move our attention from theoretical abstraction to investigating how to deal with the concrete problem we are facing, (2) let go of dogmas, (3) focus on the “cash value” of our moral intuitions, and (4) strive for inter-subjective solutions. We aim to show how these elements, grounded in pragmatist theory, can be regarded as theoretical tools and guidelines to allow people to embody a pragmatist attitude (Martela 2015) to CES. We argue that this attitude is essential for overcoming the perils of ethical pluralism in CES and is useful to improve the current dialogue in CES. Furthermore, we will demonstrate how this approach, fostering a pragmatist attitude to CES can look like in practice. For this purpose, the dilemma method, which is one of the ways of facilitating moral case deliberation (MCD) sessions, will be used as an example. We will argue that this method is in line with the four core elements of pragmatism we will elaborate and can be considered as a possible application of a pragmatist approach to CES.

In the next sections we will first describe the four core elements of a pragmatist approach to CES which are relevant to embody a pragmatist attitude. Subsequently, we will introduce the dilemma method. Next, we will reconstruct this method, pointing out how each step represents a practical application of the described core elements. We also provide a case example and end with some remarks on the strengths and limitations of this approach.

## A pragmatist answer to ethical pluralism in clinical ethics support

### Moral inquiry as a dynamic enterprise aimed at solving concrete problems

In his pivotal article “How to make our ideas clear”, Peirce argues that “the action of thought is excited by the irritation of doubt and ceases when belief is attained” (Peirce 1974a, b, p. 252): doubt arises by a hesitation in our action and stops when we are able to decide how we should act under a specific circumstance. Likewise, in a clinical environment, moral dilemmas present themselves when health care professionals doubt what choice is best in a non-trivial, urgent situation, dealing with conflicting values in unexpected situations where their acquired knowledge or moral routines are experienced as inadequate in providing guidance for the right course of action (Parker 2012). Correspondingly, Dewey argues that moral inquiry starts from a tension between conflicting values or unexpected situations “when something has to be done” (Dewey 1978, p. 169), leading to an impasse that requires concrete actions and practical solutions. Hence, according to this view, rather than theological or metaphysical consideration detached from daily life, moral reflection is instigated by and should address moral problems that people encounter in their everyday life (Dewey 1957, p. 177).

Pragmatism maintains that ethics should become a dynamic enterprise aimed at the resolution of concrete problems, and must be able to respond to the needs of a continuously changing reality rather than being a merely abstract activity in search of “immutable, extra temporal, principles, standards and norms” (Dewey 1957, xiii). As Dewey argues, every situation has its own unique “good” (Dewey 1957, p. 163) and its “specific evil” (ivi, p. 178) that need to be dealt with. Therefore, ethics should acknowledge the impossibility to find a definitive norm or principle that applies to all circumstances; it cannot be considered “a catalogue of acts nor a set of rules to be applied like drugstore prescriptions or cook-book recipes” (ivi, pp. 169–170) as “action is always specific, concrete, individualized, unique. And consequently judgments as to act to be performed must be similarly specific” (ivi, p. 167).

### ‘Unstiffening theories’: an anti-dogmatic approach

Participants in health care—professionals, patients, family members, volunteers—often experience moral dilemmas because they hold divergent views on what good care entails, and are guided by different beliefs founded in multiple religious, cultural, political or ideological backgrounds. In these cases, stakeholders not only have to face the tensions between conflicting values within one and the same framework, but also between conflicting moral outlooks on the world.

In this confrontation, the insight may arise that the knowledge or habits that were, up to that time, considered useful and efficient guides for action are no longer adequate. This happens for example when new technologies come about (i.e. genetic testing, robotic care, assisted reproductive technology etc.), enabling a whole new set of possibilities which were unconceivable before or when health care professionals are confronted for the first time with cultural beliefs or lifestyle preferences which challenge their routinary practice (i.e. refusal of sedation or blood transfusion).

According to James, when a new situation presents itself, people struggle with the search for new solutions allowing them to “marry old opinions to new facts” (James 1907, p. 61). Even though the influence of our moral intuitions and “older truths” is “absolutely controlling” (ibid.), these impasses require the capacity to look at one’s value system as such, and venture into that of other stakeholders.

To that purpose, James argues that people should be able to “unstiffen” (ivi, p. 53) their beliefs and principles, and put them into play by confronting them with the situation at hand. He argues that we should stop considering our beliefs and theories as dogmatic ideas, and start working with them in the light of an ever changing, contingent reality (ivi, p. 51). This implies admitting that moral knowledge might be fallible and may require revision. As Cooke states: “if we are open to questioning our ethical habits and beliefs, we will be open to discovering mistakes and new ethical truths” (Cooke 2003, p. 638).

Following this approach requires stakeholders to be ready to challenge former intuitions and be prepared to form new ones. According to pragmatism, moral inquiry is not a process of justification that aims at aligning facts with presupposed judgments of good and bad. On the contrary, the pragmatist attitude, requires giving up on the project of finding anything absolute and given once and for all, and demands “remaining always open to change the tools of one’s thinking, concepts, theories and so forth, to accommodate for the experiential requirements of living” (Martela 2015, p. 192).

To clarify this process, we can use Dewey’s words who argues that principles and moral intuitions must be considered as “hypotheses to be worked out in practice, and to be rejected, corrected and expanded as they fail or succeed in giving our present experience the guidance it requires” (Dewey 1957, p. 96). Instead of identifying a set of principles or norms and then applying them to particular cases, Dewey states that we should use our imagination to creatively look for new solutions. As argued by Fesmire in his book on the role of moral imagination in Dewey’s philosophy, “the point here is not that it is high time to abandon insights of traditional ethical theories. To the contrary, it is time to return to these traditions with an eye to reconstructing their troublesome elements” (Fesmire 2003, p. 60). This means that the principles we use to guide our conduct are not “dogmas” (Dewey 1957, p. 96), but “instruments” (James 1907, p. 58) that must demonstrate their usefulness and their practical applicability.

In this context, long-established norms traditionally accepted within a mono-cultural community, are not to be rejected as such, but they have to be adapted, “expanded and corrected” (Dewey 1957, p. 96) in order to do justice to the requests of recognition of different stakeholders and to the needs of a continuously changing reality (Dewey 1978, p. 226). In this line.CES in a pluralistic setting should therefore focus on finding ways to conjugate divergent perspectives in order to provide practical and shared solutions while still respecting the ethical differences at stake. In other words, we should avoid considering our beliefs and theories as fixed frameworks, and start using them as “tools” to build new solutions together (Miller et al. 1996, p. 130; Fins et al. 1998, p. 40). In this way, conflicting values and theories are not discarded as such, since they represent the essential starting point of every moral inquiry, but they are addressed with an open and experimental attitude, as scientific hypothesis to be applied and verified time and again.

### Focus on the “cash value” of principles and moral intuitions

What makes our theories and beliefs valuable to us? According to James, answering this question means finding out what is their “cash value” (James 1907, p. 53) , and establish what is their practical meaning, i.e. the action, behaviour or understanding caused by them.

In order to understand the practical meaning and the “truth value” of our beliefs and principles we need to elucidate what effect “truths” or moral habits have on our lives in moral terms. What do my moral principles mean in practical terms? Which concrete action follow from them? How do my moral intuitions guide my actions, and therefore determine a change in my world and my interaction with it? “In what respect would the world be different if this alternative or that were true” (James 1907, p. 48)? In more general terms, Pierce claims that we should “consider what effects, that might conceivably have practical bearings, we conceive the object of our conception to have. Then, our conception of these effects is the whole of our conception of the object” (Peirce 1974a, b, p. 258).

In providing CES, applying this maxim allows stakeholders to avoid abstract debates and stimulates them to explore the practical consequences of their moral intuitions. A pragmatist approach to CES aims to interpret “each notion by tracing its respective practical consequences” (James 1907, p. 45). James states that truth is always made through experience; true thoughts are those able to guide us, because they represent valid instruments for action. They “agree” (ivi, p. 212) with reality in the sense that they help us to find a way of acting that is in accordance with both our beliefs and concrete facts. In fact, both James and Dewey concur that true thoughts do not reflect reality as a “mirror” (Rorty 1979) but guide the subject (Dewey 1957, p. 156) by providing valid instruments to support the action, and by representing reliable mental means of adaptation to reality. This ability to guide us is their “truth value”. To quote Dewey:

If ideas, meanings, conceptions, notions, theories, systems are instrumental to an active reorganization of the given environment, to a removal of some specific trouble or perplexity, then the test of their validity and value lies in accomplishing this work. If they succeed in their office, they are reliable, sound, valid, good, true (ibid.).

Therefore, the pragmatist approach invites us to find out what is the “cash value” of our moral intuitions, and this means turning away from the abstractness of our theories and looking into “facts”, “concreteness”, and “adequacy” (ivi, p. 51) in order to clarify how our theories are useful in removing the specific “evil” entailed in the situation at stake.

### Morality as inter-subjective activity

According to American pragmatism, focusing on the concreteness of the situation at stake also means accepting the impossibility of reaching a “God perspective” (Bernstein 2010), i.e. an external and objective point of view. We will always have to depart from our experiences, perspectives, and divergent values that influence the way a situation is regarded. This implies that there is no common reference point among individuals, but rather a plethora of ways of looking at and experiencing the world. Hence, a morality based on an alleged consensus that denies fundamental interpersonal differences in the way in which a moral issue is approached, will fail in handling the challenges presented in a pluralist society, instead of constructively face them. In a pragmatist approach, moral disagreement is not only seen as unavoidable, but also as constructive, since it encourages us to engage in a shared process of inquiry in which people are forced to consider and to become aware of the relationship between what is considered to be “good” by a social group and what is considered “good” by the single individual (Dewey 1978, p. 198). In fact, both Dewey and Peirce underline the social and inter-subjective nature of knowledge. Peirce argues that in order to determine what the best opinions are, we have to subject them to a continuous process of inter-subjective verification (Peirce 1974a, b, p. 235). This means giving up on absoluteness but not on objectivity (Rorty 1993). Absolutists argue that the goal of this inter-subjective process is to find a way to end the conversation by finding an agreement and conclusion, which makes further debate unnecessary (Rorty 1982, p. 170). On the contrary, pragmatists argue the opposite, as we do not have any “metaphysical or epistemological guarantee of success […] we do not know what ‘success’ would mean except simply ‘continuance’” (ibid.). This means that the dialogue and conversation, following democratic standards, have both an instrumental and an intrinsic value, and represent the structure and process required to achieve moral objectivity (Cooke 2003, p. 645). According to Dewey, to universalize does not mean finding an absolute norm, “universalization means socialization” (Dewey 1957, p. 206). This does not mean that pragmatists give up on objectivity. According to Hester, “objectivity is taken in an operative and not ontological sense” (Hester 2003, p. 550). It is always contextual, inter-subjective and resulting from a shared process of inquiry conducted within a democratic framework. As a result, the adequacy of our thoughts is always context-bound and related to its ability to guide the action in accordance with the social, cultural, emotional and factual situation in which it is embedded (James 1907, pp. 215–217). By emphasizing the operative nature of objectivity, and moving away from an ontological approach, the pragmatist approach is distinguished from other dialogical approaches to ethics, such as the intercultural dialogues developed by religious institutions, which tend to regard certain values and normative starting points as given.

In the following sections, we will demonstrate that a method to provide concrete ethics support can be in line with a pragmatist approach as outlined above. We will do so by reconstructing MCD, the dilemma method in particular, in terms of the elements of a pragmatist approach. To this purpose, we will first provide a brief introduction to MCD and then discuss the dilemma method step by step, showing how the four elements of what could be a pragmatist approach to CES are integrated in each step.

## Moral case deliberation as pragmatist clinical ethics support

MCD, a form of CES widely used in the Netherlands and increasingly popular elsewhere in Europe, consists of multidisciplinary meetings involving health care professionals, including nurses, physicians, social workers, but also managers families and patients (Stolper et al. 2016), aiming to engage in a dialogue about a concrete case directly experienced by participants as morally troublesome. It is a structured dialogue rather than a decision making method, aiming to guide a shared self-reflection process that allows professionals to explore the moral dimension of the situation at stake by making appeal on values, feelings and personal emotions (Dauwerse et al. 2014; Weidema et al. 2013; Molewijk et al. 2011).

The primary goals of MCD are: (1) to reflect on the case and on the quality of care in relation to a specific case, (2) to reflect on what it means to be a good professional, (3) to improve moral competencies and (4) to reflect on the moral quality of care at an institutional or organizational level (Abma et al. 2009, p. 219). The sessions are guided by a clinical ethicist or a trained facilitator; her role is to foster the dialogue among participants in order to help them to learn “from experience through dialogue with others” (Abma et al. 2010, p. 244). This means that she does not act as a teacher or an “answer giver”, she is not meant to advise people on their choice of action, but to focus “on the process by which the group members reach this answer on their own” (Molewijk et al. 2008a, p. 44). Therefore, the facilitator should structure the dialogue by helping stakeholders clarify the moral content emerging from the evaluation of the case, with the intention of improving their moral awareness (Molewijk et al. 2008b) and helping them gain a better insight on the ethical dimension of their practice.

The moral issue in question can vary depending on circumstances; for example, in psychiatric hospitals, MCD can focus on issues around seclusion, assisted suicide, or care time-management (ivi, p. 47; Molewijk et al. 2008c, pp. 58–59). The facilitator can use different conversation methods in accordance with the aim of the session. In this paper we focus on the so called dilemma method (Stolper et al. 2016). By following this specific conversation method, the facilitator helps the stakeholders to point out the moral dilemma they face and which moral issues are related to that dilemma. The dialogue starts from the concrete experience of participants, and supports them in reflecting on this experience. During the meeting, the ethical expertise of participants is made explicit, and enhanced through dialogue. This focus on concrete experience is a crucial pragmatist aspect of the method, however each step carries elements of a pragmatist approach. Therefore, in the following section we will explore the method with the intention of highlighting how it embodies the four elements described.

## The steps of the ‘dilemma method’ in light of pragmatism

The dilemma method is a type of MCD, in which the joint reflection process is structured in various steps (Stolper et al. 2012, pp. 57–58, 2016; Molewijk and Ahlzen 2011, pp. 9–10). In Table 1, we will relate each step to the elements of a pragmatist approach to CES.

**Table 1:** Dilemma method: overview

The dilemma method: structure of the reflection process
Step 1. Introduction—determination of goals and expectations and explanation of the method
Step 2. Presentation of the case by one of the participants
Step 3. Formulation of the dilemma and of the moral questions related to it
Step 4. Clarification and empathizing—participants put themselves in the shoes of the case presenter
Step 5. Analysis of stakeholders’ perspectives, values and norms
Step 6. Looking for alternatives (both feasible and not feasible)
Step 7. Making and motivating individual choices and considerations
Step 8. Dialogical inquiry—analysis of similarities and differences between individual choices
Step 9. Conclusion—answering moral questions and looking for shared decision (if necessary)

### Step 1. Introduction: anti-dogmatic and practice oriented approach

The facilitator begins the session by giving a brief overview of the steps (if the participants are not yet familiar with the method) and by asking the participants what they expect and wish to establish with the deliberation. The aim is not decided in advance but it is determined by participants, in accordance to their needs. In line with Dewey’s recommendation, moral inquiry in MCD starts with the recognition of a concrete issue that participants are dealing with in a specific case (Dewey 1957, pp. 165–166).

The facilitator underlines that the aim of the session is not to give answers or provide solutions on the basis of theoretical knowledge (Dewey 1957, pp. 169–170) but to reason on a concrete problem by appealing to everyday experiences and moral intuitions. Starting from the Deweyan assumption that ethics can never be considered as the mere application of presupposed rules and intuitions (ivi, pp. 169–170), the facilitator urges participants to avoid giving quick answers and postpone their initial judgments, by assuming and open attitude towards the case which will be discussed. Moral intuitions, values and perspectives are therefore treated as hypotheses that, as Dewey argues, have to be intended as instruments, which can be changed or abandoned if they fail in guiding the action to the resolution of problems that have to be faced (Dewey 1957, p. 96, pp. 145–6). Hence, the participants are invited to consider others’ as well as their own moral intuitions in an anti-dogmatic way (Dewey 1957, p. 96) allowing them to “unstiffen” them and work with them to reach a shared decision (if that is the goal of the meeting).

### Step 2. Presentation of the case: facts and experience as the starting point of moral reflection

In the second step, the case presenter, who is personally involved in the situation at stake, explains the case, elaborating on the aspects that made it morally troublesome for her, by referring to her personal feelings, values and thoughts. It is important for the case presenter to keep an open attitude and not to jump to conclusions. However, this does not mean the description should be impersonal or objective. Rather, the focus is on “lived experience”, i.e. one’s personal and situated concrete experience of a situation (James 1907, pp. 6–8, 1956, p. 11; Dewey 1933, p. 28). The facilitator encourages the presenter not only to describe the course of events meticulously, but also to focus on the moment in which she perceived the problem to be urgent and pressing, or, in other words, the moment in which moral doubt arose.

According to Dewey, reasoning should start with observation, because observing encourages you to include concrete facts in the inquiry process and to consider the unicity of a particular situation (Dewey 1957, p. 140). What is shown as adequate in one circumstance may not be adequate in another. Therefore, one should avoid abstract discourse on principles and abstract theories, and direct attention to the concreteness of the specific problem (ivi, pp. 165–166).

### Step 3. Articulation of the dilemma: understanding “concrete evils” and focusing on consequences

The facilitator helps the case presenter articulate the moral issue in terms of a moral dilemma, i.e. a choice between two different courses of action that are mutually exclusive, and both of which have disadvantages (hence, the common description of a moral dilemma as “a choice between two evils”). If the dilemma is unclear, the facilitator helps the case presenter focus on the moment in which she perceived the problem to be the most prominent and in which she was facing the question: should I do A or should I do B? If there are more than two options, the facilitator methodically focuses on the two most important ones, in order to find a point of departure for the joint reflection. Other potential options can be considered in a later step.

After the articulation of the dilemma, the participants are invited to identify possible negative consequences of both choices. From a pragmatist point of view, this exercise has two important functions. Firstly, specifying the consequences of the two options means applying Peirce’s pragmatic maxim, which entails that in order to understand the meaning of an idea we should understand what its practical consequences are. Secondly, this process enables participants to point out the concrete “evils” which are to be removed (Dewey 1957, p. 177) and helps them to keep focused on the concrete, and the “factual importance” of the dilemma.

### Step 4. Clarification of the facts in the case: understanding the concrete doubts and empathizing

In this step, participants ask specific questions to the case presenter, in order to clarify facts, emotions and considerations that might be relevant for putting themselves in her shoes. As already mentioned, in this method participants focus on situations which Peirce would define as “living doubts” (Peirce 1974a, b, p. 229), in which stakeholders need to face the fallibility of their presupposition and are confronted with a concrete and unavoidable impasse, which cannot be detached from the practical problem from which it arose. The doubt that the case presenter faces is radically concrete and comes with a sense of “struggle”, which urges stakeholders to reach a practical solution. In order for participants to engage in the deliberation they have to “feel” the living doubt, and understand the tension entailed in the situation at stake. Thus, in this step, the case presenter is asked to point out situational information, feelings, fears, and impressions that she might have left out while presenting the case, but which might be important for other participants. The aim of this step is to clarify the details of the case especially those that make the situation doubtful and morally troublesome.

### Step 5. Analysis of perspectives, values and norms: “cash value” and plurality of views

In the fifth step, participants investigate important values of all the stakeholders (perspectives). For each perspective, values relevant to the dilemma are identified, and concretised in specific norms or action rules. In order to do so, participants are asked to answer the following question: “what should be done in light of this value?”, “what behaviour would follow from this value for this person (perspective)” or “what rule of action would follow from the value in this situation”? For instance participants can name “care” as a value and phrase the corresponding norm as “I should always take the patients values into account when suggesting a treatment plan”. Same values can translate into different norms and vice versa the same norm can relate to different values.

This analysis represents two of the core elements discussed before. Firstly, participants apply the pragmatic maxim and make explicit the “cash value” of principles and moral intuitions; values are transformed in norms of action, thereby allowing participants to see what are the practical consequences of the values at stake. In this way the dilemma method flattens theoretical disputes and brings the participants’ attention back to the level of facts (James 1907, p. 51) by concretely showing them which concrete rule of action follows from their values. Secondly, this step allows participants to analyse the values of all the subjects involved. Therefore, rather than suppressing the conflict between different perspectives, this analysis allows the participants to explore these differences, by making them visible and therefore comprehensible. Moreover, by clarifying the meaning of each moral perspective in practice, participants might reach broader understanding of what concepts like “autonomy” or “respect” mean concretely in the case at stake, which leads to the discovery or refinement of what acting upon those concepts means in the concrete situations.

The goal of this step is not to reach an objective or comprehensive understanding, but a situated one. According to Rorty, when in doubt about what to do, we should provide a new description of the problematic situation we are facing (Rorty 1989, p. 78) in order to include all the different interpretations of it. In doing so we “enlarge the canon” of our comprehension (ivi, p. 81) and provide a broader description of the situation that can be helpful to clarify what moral issues are at stake. By making differences explicit, and by focusing on what those differences could mean in practice, MCD realises one of the pragmatists goals, and fills the gaps between moral intuitions and practice.

### Step 6. Looking for alternatives: creatively rethinking the situation

In this phase participants are invited to consider possible alternative actions, other than the two courses of action articulated in the central moral dilemma. The aim of this exercise is both to escape from the two evils entailed in the moral dilemma by creatively exploring other alternative solutions. The focus in on possible actions which might be realistic or not. In this step participants are asked to use their moral imagination and creatively think of a solution which was not explored before, once again escaping from their preconceptions and exploring the situation by taking an anti-dogmatic approach.

### Step 7. Individual choices and considerations: the “practical meaning” of moral judgments

In this step, participants make an individual choice; they express what their decision would be if they were in the position of the person who has to decide between the two options. The decision needs to be justified, and each participant has to indicate which values underlie their judgement with concern to the best course of action, and which other values are given less priority. Furthermore, they are asked what they would need to implement their choice, and to consider possible disadvantages of it, and how they could be dealt with. In this way, participants take into account the practical consequences of their choices in their moral judgment.

Hence, the justification of their individual weighing of the moral dilemma is not limited to the consideration of the moral values that are guiding their decision, but has to be accompanied by an evaluation of the means that are necessary to translate the choice into practice, and by a consideration of the negative consequences that could also follow from their choice for a certain course of action. Thus, moral deliberation starts with practice, and goes back to practice; moral intuitions and judgments are never considered per se but always as instruments to be used to act upon a certain problem. In line with Dewey, moral reasoning should lead to the achievement of practical solutions for concrete problems encountered by specific individuals. In this step this goal is pursued, and each moral judgment expressed by participants is translated in an actionable decision, which should lead to the resolution of the practical issues at stake.

### Step 8. Dialogical inquiry: looking for intersubjective understanding

In this step, participants explore the similarities and differences between their choices and underlying moral reasoning. By investigating differences and similarities, participants learn to see the relevance of other perspectives for the evaluation of the situation. In line with pragmatism, no one can find “truth” alone; inquiry is always a social activity in which different subjects are called upon to collaborate in order to shape a shared understanding of the world. This corresponds to the Deweyan idea that “universalization means socialization” (Dewey 1957, p. 206): in order to find a solution to a moral problem we have to broaden each personal perspective by confronting it with those of others. However, this is not about converging all individual perspectives in a comprehensive and definitive consensus; on the contrary, we should look for an inter-subjective understanding that acknowledges different perspectives instead of ignoring them (Rorty 1991, p. 23). Likewise, this step aims to build a common understanding in which disagreement among stakeholders is taken into account as a positive stimulus to develop new insights and new awareness.

### Step 9. Conclusion: a “marriage function” between old truths and new facts

Finally, participants are invited to come to conclusions. They try to answer the central moral question and (if possible) look for a shared agreement on how to act. From a pragmatist perspective, the choice will be considered appropriate if it guides action in accordance with divergent instances that characterize the case, thereby realizing a “marriage function” (James 1907, p. 64) between old values and new situations. Indeed, according to Dewey and James the ability to guide action and realize a mediation and a reconciliation between different beliefs or between new problems and “old” beliefs will represent the “truth value” of the solution and will determine its success (ivi, p. 61). In MCD this goal is achieved through dialogue. Consensus (although preferable) is not the main goal and it is not the only outcome of moral reasoning. In fact it is desirable that the “[a]greement reached through compromise and through consensus leads to transformation in understanding” (Cooke 2003, p. 649). Following James, while looking for solutions to troublesome situation, we should try to preserve “the older stock of truths with a minimum of modification, starching them just enough to make them admit the novelty, but conceiving that in ways as familiar as the case leaves possible” (ivi, p. 60). Therefore consensus is not only important for the outcomes of inquiry but it is “a continuum of process and outcomes known as intelligent inquiry itself” (Hester 2003, p. 551). The aim is to reach a conclusion that can be workable for stakeholders and that does not represent a violent imposition or a revolutionary statement. This means that in order to be accepted, new solutions have to be integrated in the pre-existing set of beliefs and principles that characterizes the moral environment in which the case takes place.

## Example

In order to better understand this process we will briefly present an MCD described in an article by Tan et al. (2018)^1^. A Moroccan-Dutch 32 year old male rugby player collapsed during a game. After two resuscitation procedures and poor responses, after 2 days he had a “spontaneous ventilatory drive, a Glasgow Coma Scale score of E1 M2 V1 and equal pupils that were unresponsive to light” (ivi, p. 182). Given the poor neurologic prognosis, the ward’s team started considering withdrawing treatment. However, this suggestion was not welcomed by the relatives of the patient, who, out of religious believes, wanted treatment to be continue, in order to keep him alive. According to their faith only Allah decides when life should be ended. The neurology resident, who presented the case during the MCD, was confronted with a dilemma: ‘Should I continue treatment (A) or should I stop treatment (i.e., remove tracheal tube and supportive medication) (B)?’ (ibid.). In this case the team was confronted with a clash of values: on one hand, most of the team agreed that prolonging treatment would be medically useless, and not in the best interest of the patient. On the other hand, the team also wanted to respect the values of the patient and have a harmonious relationship with his family. Through dialogue, participants identified the core values and points of agreement and disagreement within the team. Most team members thought stopping treatment was the right thing to do, although this would upset the family. One of the nurses wanted to continue treatment, while explaining to the family that this would in the end not save the patient’s life. Another participants suggested to consult the hospital’s intercultural healthcare professional and engage in a dialogue with the family to reach a mutual understanding that “the eventual decision should be in the best interest of the patient, both from his perspective […] and the medical perspective” (ivi, p. 184). As a result of the MCD, it was decided to stop treatment and discuss this with the family members in the presence of an intercultural health care consultant. This conclusion was based on providing good and efficient medical care while trying to maintain a harmonious relation with the family members.

The reflection helped the participants in identifying the core issue at stake, and in acknowledging the need for further understanding and elaboration on “what does it mean in this specific situation to pursue the best interest of the patient?”. On the one hand, the best interest of the patient appeared to be to stop—a conclusion which could also have been argued by following a principlist approach, as continuing treatment would not be beneficial, and cause harm. On the other hand, acting in the best interest of the patient was seen as taking into account his relational context, and fostering a peaceful end of his life in a harmonious situation. Thus, the notion of best interest changed, by a process of unstiffening of existing theories about best interest, and taking into account new facts, resulting in new joint understanding of all the team members, responding to the family perspective.

## Discussion

It should be underlined that the pragmatist approach presented does presuppose that all participants (both stakeholders and facilitators or clinical ethicists) are willing to embody a certain attitude, or in other words, to be pragmatists themselves. This assumption is not self-evident, and, within our pluralist society, people (and also stakeholders in clinical practice) may be unwilling to do so. For instance participants may seek to adhere to principles without ‘unstiffening’ them. The four elements we describe are instrumental to foster such pragmatist attitude, which allow people to address issues in which ethical pluralism is at stake. As both Dewey and James argue, pragmatism is primarily a method and an attitude (Martela 2015, p. 188). It is per se compatible with different theories but it requires participants to (1) be willing to use their theories as tools, by acknowledging their usability (cash value) and possible fallibility (ivi, p. 194) and (2) engage in a process of shared and inter-subjective inquiry, following democratic rules.

Some authors have raised doubts about this approach: what good reason do we have to change our belief? How do we ensure that by starting to question our fundamental principles we will not fall into relativism and anti-realism (Tollefsen and Cherry 2003)? This questions have been addressed among others by Hester (2003), Rorty (1993) and Putnam (1995), who argue that pragmatism is not incompatible with objectivity and is able to resist the accusations of relativism. Take the example in the case presented above. A definitive understanding of the best interest of the patient cannot be reached, as different theoretical points of view clash; the individualist and the relational interpretation of the patient’s interest are at odds. Putting forward the individualist approach, which the team was inclined to do, does not help in solving the issue with the family. In such situations, no progress can be made if stakeholders are not willing to engage in a dialogue with an open and anti-dogmatic attitude in order to find shared and morally sound decisions. If stakeholders embody a pragmatist attitude, and are willing to engage in a shared reflection with and anti-dogmatic look, a new view on the best interest of the patient can be found, which is not relativistic, since it is not a matter of ‘anything goes’, but is fitted to the situation and enables solving the problem.

Being anti-dogmatic towards one’s own moral presuppositions does not mean that participants should start from the position of doubting everything (as Descartes would suggest). In fact, as Putnam argues “the participants to an actual discussion always share a large number of both factual assumptions and value assumptions that are not in question in the specific dispute. Very often the disagreement can be resolved not by appeal to universal set of criteria but to values which are not in question in that disagreement” (Putnam 1995, p. 22). Given the willingness to find a solution to a specific issue, participants can build up consensus by unstiffening incompatible theories, while finding a way to be directed by the normativity of those shared values which are not questioned. In this way, the anti-dogmatic approach gets balanced by the  willingness to reach consensus and realize a marriage function between new challenges and old truths.

In a pragmatist approach CES participants and providers should assume the attitude of wanting to “accommodate for the experimental requirement of living” (Martela 2015, p. 193). Although such an attitude cannot be enforced, it can be fostered by providing certain conditions for deliberation. In MCD, specific tools are available for creating an open atmosphere, and inviting participants to listen to each other and postpone their own moral convictions. These tools include focusing on the facts of the situation, urging participants to transform opinions into questions, and enabling all participants to present their own perspective (Molewijk et al. 2008b, c). In the end, in line with this attitude, CES, based on the dilemma method, does not aim to provide universal answers valid in all circumstances, but focuses on the specific and concrete problems that arise in a peculiar context and ask for particular solutions. The goal of this method is not to provide theoretical notions but to develop stakeholders’ critical awareness, which can be useful in their professional work. In this way, caregivers learn how to change without giving up their convictions, by modifying and unstiffening them, in order to adapt them to their interlocutors’ demand for recognition. Therefore, the outcomes of moral deliberation will be adequate if they have a practical value; their “truth value” will not rest on the absoluteness of its presupposition, but on the possibility to form a valid solution for the problem at stake, and on its ability to realize a “marriage function” between divergent positions.

Fostering an open dialogue implies a specific role of the ethicist. The ethicist should act as a facilitator, inviting participants to ask questions and enter into a joint process of investigation. Instead of falling back on theoretical knowledge, the facilitator should focus on making explicit practical insights of the participants. The steps of the dilemma method may support the process of facilitation. Yet, they cannot be applied in a technical way, but require practical knowledge and skills on the side of the facilitator who should be able to foster dialogue, to manage conflict resolution and to value the experiential knowledge of participants. The capacities required for facilitating MCD cannot be learned by the book alone, and do not have a pure philosophical nature, but require practical training. A pragmatist approach to CES in general, and MCD in particular, requires exercise and “learning by doing” (Stolper et al. 2015). Thus, the theoretical tools of philosophical pragmatism and their elaboration in the steps of the dilemma method, are only relevant for CES if they inform practice and are embodied in the routines of the professionals involved in the practice of CES.

Interestingly, some critiques argue that bioethics is inherently pragmatist (Tollefsen 2000; Arras 2002, p. 33; Schmidt-Felzmann 2003; Moreno 2003). Among others Arras claims that American bioethics has always tried to avoid the reference to “ground philosophical schemas in favour of pragmatic policy making and democratic consensus” (Arras 2002, p. 29). In particular it may be argued that the pragmatist approach resembles “specified principlism”, which, inspired by the Rawlsian idea of reflective equilibrium, tries to provide guidance for those situations in which different principles conflict (Tollefsen 2000; Arras 2002, p. 33; Schmidt-Felzmann 2003; Moreno 2003). Specified principlism, by focusing on the “mutual support of the whole set of norms” (Richardson 1990, p. 299), aims at safeguarding and enhancing the coherence of the given set of principles and norms derived from them, while providing guidance on how to justify concrete choices in case of conflict. Yet, in spite of similarities with specified principlism, pragmatism is more radical, in claiming that principles have only a functional and not fundamental value (Hester 2003, p. 554). In contrast with specified principlism, a pragmatist approach entails that principles must arise from specific problems, and not be referred to in order to justify moral judgments and actions. They “evolve over time” (Mallia and ten Have 2005, p. 71) and their guidance is binding until something contradictory happens, or until the moral routine is challenged and presupposed principles do not succeed anymore in guiding the practice.

## Conclusions

Can we provide CES without shared foundational moral principles? We believe we can by using a pragmatist approach as described in this paper. Indeed, a pragmatist approach to CES provides us with a way to deal with moral pluralism because it allows stakeholders to work with their moral intuitions in an anti-dogmatic way; personal convictions are neither neglected nor discarded, but used as instruments to find practical solutions. The dilemma method embodies a pragmatist approach to CES because it: (1) fosters a focus on facts, actions, and considerations that are useful to understand why the situation is morally troublesome, and by pointing out the concrete evil we need to face; (2) enables participants to share their values and moral beliefs by assuming an open attitude that will allow them to work with their convictions in an anti-dogmatic way; (3) urges participants to focus on the cash value of their moral intuitions and values; (4) fosters a shared process of moral inquiry that looks for a solution which is embedded in the emotional and factual reality that generates the dilemma and actually works in practice.

This approach works best in a context in which diversity is the starting point, and in which doubts, or frictions between presupposed beliefs and new challenges (being those different beliefs or new situations) occur. In these situations a pragmatist attitude is required. Practicing a pragmatist attitude means embodying the four elements just described and stop looking at values and norms as abstract and dogmatic rules, which cannot be adjusted, to start reflecting on how they can be useful to solve the specific moral problems we encounter in clinical practice. This provides us with a way out of the impasse faced in a pluralistic setting, i.e. having to choose between either objectivism or radical relativism—or even moral skepticism.

According to this approach, being anti-dogmatic and ready to revise given theories and practices is what sets moral reasoning in motion, and the driving force behind moral progress. In the same line, both the four elements described and MCD should be seen as tools, rather than as fixed rules or methods for action that are valid per se, which should be modified in case they do not meet the needs of the situation at stake.

## Footnotes

1 For more case examples, see Widdershoven et al. (2014), Stolper et al. (2016), Voskes et al. (2016), Molewijk and Ahlzen (2011), and Metselaar et al. (2017).
